# Patients, healthcare providers, and general population preferences for hemodialysis vascular access: a discrete choice experiment

**DOI:** 10.3389/fpubh.2024.1047769

**Published:** 2024-05-09

**Authors:** Tak-Sui Wong, Qian Chen, Taoran Liu, Jing Yu, Yangyang Gao, Yan He, Qiongqiong Zhong, Zijian Tan, Tinlun Liu, Jian Lu, Jian Huang, Casper J. P. Zhang, Lianghong Yin, Bo Hu, Wai-Kit Ming

**Affiliations:** ^1^Division of Nephrology, Department of Medicine, The First Affiliated Hospital, Jinan University, Guangzhou, China; ^2^Department of Infectious Diseases and Public Health, Jockey Club College of Veterinary Medicine and Life Sciences, City University of Hong Kong, Kowloon, Hong Kong SAR, China; ^3^Department of Public Health and Preventive Medicine, School of Medicine, Jinan University, Guangzhou, China; ^4^International School, Jinan University, Guangzhou, China; ^5^Singapore Institute for Clinical Sciences (SICS), Agency for Science, Technology and Research (A*STAR), Singapore, Singapore; ^6^Bioinformatics Institute, Agency for Science, Technology and Research (A*STAR), Singapore, Singapore; ^7^Department of Epidemiology and Biostatistics, School of Public Health, Imperial College London, Norfolk Place, London, United Kingdom; ^8^School of Public Health, Li Ka Shing Faculty of Medicine, The University of Hong Kong, Pokfulam, Hong Kong SAR, China

**Keywords:** patients, healthcare providers, general population, hemodialysis, vascular access, discrete choice experiment, preference

## Abstract

**Background:**

A patient-centered dialysis treatment option requires an understanding of patient preferences for alternative vascular accesses and nephrologists often face difficulties when recommending vascular access to end-stage kidney disease (ESKD) patients. We aimed to quantify the relative importance of various vascular access characteristics to patients, healthcare providers and general population, and how they affect acceptability for patients and healthcare providers.

**Methods:**

In a discrete choice experiment, patients with maintenance hemodialysis (MHD), healthcare providers, and individuals from the general population were invited to respond to a series of hypothetical vascular access scenarios that differed in five attributes: cumulative patency, infection rate, thrombosis rate, cost, and time to maturation. We estimated the respondents’ preference heterogeneity and relative importance of the attributes with a mixed logit model (MXL) and predicted the willingness to pay (WTP) of respondents via a multinomial logit model (MNL).

**Results:**

Healthcare providers (*n* = 316) and the general population (*n* = 268) exhibited a favorable inclination toward longer cumulative patency, lower access infection rate and lower access thrombosis rate. In contrast, the patients (*n* = 253) showed a preference for a 3-year cumulative patency, 8% access infection rate, 35% access thrombosis rate and 1.5 access maturity time, with only the 3-year cumulative patency reaching statistical significance. Among the three respondent groups, the general population found cumulative patency less important than healthcare providers and patients did. Patients demonstrated the highest WTP for cumulative patency, indicating a willingness to pay an extra RMB$24,720(US$3,708) for each additional year of patency time.

**Conclusion:**

Patients and healthcare providers had a strong preference for vascular access with superior patency. While the general population preferred vascular access with lower thrombosis rates. These results indicate that most patients prefer autogenous arteriovenous fistula (AVF) as an appropriate choice for vascular access due to its superior patency and lower complications than other vascular access types.

## Introduction

Chronic kidney disease (CKD) comprises one of the main causes of global morbidity and mortality (1). In recent years, the aging population and the growing incidence of diseases, such as diabetes and hypertension, have led to an increase in CKD incidence by years (2). The global prevalence rate of CKD in the general population has reached 14.3% (3). In China, the prevalence of CKD in patients over 18 years old was 8.2, and 1.8% of patients progress to end-stage kidney disease (ESKD) each year (4). Thus, renal replacement therapy is needed, mainly hemodialysis (HD), peritoneal dialysis (PD), or kidney transplantation (5). Maintenance hemodialysis (MHD) is a treatment to prolong the life of ESKD patients through regular hemodialysis (6). According to the Chinese National Renal Data System (CNRDS), there were approximately 0.63 million HD patients in China in 2019, and the high cost of treatment imposed a huge financial burden on the families of patients and society (2). During the hemodialysis, vascular access is used to transfer blood between the body and the dialysis machine. Morbidity related to vascular access is the leading cause of hospitalization for patients with MHD, the development of complications is the main reason why patients become disillusioned with hemodialysis therapy (7).

Among permanent hemodialysis vascular access, autogenous arteriovenous fistula (AVF), arteriovenous graft (AVG), and tunneled-cuffed catheter (TCC) are widely used, with various discrepancies in dimensions of cumulative patency, infection rate, cost, etc. Previous practice guidelines and initiatives have identified AVF as the best option associated with its better patency and lower complications than other vascular access types (8, 9). However, the strengths of AVF may be overestimated, as a high proportion of AVFs fail to mature successfully before it can be used, and interventions are commonly needed to promote maturation (10–14). Applying temporary catheters during AVF non-maturation time increases the risk of patient exposure to infection, leading to bacteremia and thus endangering the patient’s life (15, 16). AVG has been proven to be a suitable alternative of AVF creation for patients due to poor vascular conditions, but this access type’s long-term failure and intervention rates are higher than those of AVF (17, 18). Accordingly, nephrologists often face difficulties when making dialysis-related decisions for ESKD patients regarding life expectancy, patients’ anatomy, and the associated complications with vascular access types. In clinical practice, doctors and patients have different main considerations when choosing vascular access for hemodialysis. Specifically, clinicians are concerned about the vascular access type and its associated complications. However, patients may have far differing concerns (19, 20), such as the pain and fear of needle and physical disfigurement from an AVF (7), which makes it necessary to understand the rationale behind both options to facilitate transparent shared decision-making, and may further help doctors understand the idiosyncrasies and demands of patients and their families, and choose more appropriate vascular access for patients, which is of vital importance for reducing potential doctor-patient conflicts and general improved patient satisfaction. In this study, the preferences of the general population were used as a reference. In addition, the preferences of the general population can be surveyed as a reference to better understand patient preferences.

Discrete choice experiments (DCEs) have been widely used to assess healthcare priorities, mainly for selecting therapeutic drugs and protocols (21). This approach simulates real-world decisions by simultaneously considering multiple characteristics, thus determining the strength of preferences, including CKD patients regarding organ donation and end-of-life care (22). To our knowledge, no published studies have been conducted on the use of DCE in the selection of vascular access for hemodialysis. Therefore, we aimed to use DCE to simulate clinical conditions to determine the relative influence of various characteristics on nephrologists’ recommendations and to explore which factors patients and their families are most sensitive to hemodialysis vascular access, and to quantify the preferences of patients when seeking medical treatments, and to further provide a guideline for doctors to make an appropriate hemodialysis vascular access plan for each patient.

## Methods

### Attributes and levels design

Discrete choice experiments (DCEs) are experimental designs that typically be used to quantify and weigh the relative importance that patients place on various treatment attributes and outcomes. Discrete choice experiments are based on the multi-attribute utility theory in economics (23), which assumes that commodities consist of a series of attributes and levels, such as treatment methods and expenses. In this experiment, respondents were presented with a sequence of questions and asked to select a preferred option from a set of hypothetical treatment options. These dialysis treatment protocols vary by treatment attributes and levels. The implementation of DCE in this study is in accordance with the ISPOR (International Society for Pharmacoeconomics and Outcomes Research) report (24, 25).

The attributes and the levels were, respectively, determined through a targeted literature review and discussion with experts. We compiled a list of potential attributes from previous studies in the field of hemodialysis over the past decade that included published hemodialysis DCE studies (22, 26, 27), clinical relevant articles (6, 12, 14, 16, 17, 28–32), observational studies (20, 33, 34), systematic review (7). A focus group meeting was then held at Jinan University’s Affiliated Hospital to further determine the attribute list, which involved two attending nephrologists with 7 and 8 years of clinical experience, respectively, and three patients with hemodialysis. Based on their preferences, we ranked the attributes from most important to least important. To reduce the cognitive burden on the subjects, we selected the five most relevant attributes from the ranked results (Table 1), because attributes ranked sixth or more were considered less important by experts and patients. Five attributes were finally determined in DCE, specifically: cumulative patency, infection rate, thrombosis rate, time to maturation, and cost. The minimum and maximum levels of the five attributes were determined based on literature review (28, 29, 31, 32) of clinical data resources, characteristics of vascular access, and experts’ opinions. The intermediate level was determined by calculating the median value between the minimum and maximum levels. Each attribute has three corresponding levels. Details of the identified attributes and levels are shown in Table 1.

**Table 1:** Attributes and levels in the DCE.

Attributes	Definition	Levels of Attributes (regression coding)
Cumulative patency	Time from vascular access creation or insertion (central venous catheter) to permanent failure	1 year
		3 years
		5 years
Access infection rate	The occurrence of any infection involving the vascular access	1%
		8%
		15%
Access thrombosis rate	The occurrence of thrombotic occlusion of vascular access	20%
		35%
		50%
Time to maturation	The time from access placement to its successful use for dialysis	0 month
		1.5 months
		3 months
Access creation cost	The total hospitalization cost, including operation fee, physician fee, cost of investigations, cost of supplies and interim dialysis sessions	¥ 10,000
		¥ 25,000
		¥ 40,000

¥, Chinese yuan (1 ¥ ≈ 0.15US dollars).

### Participants

At the hemodialysis center of Jinan University Affiliated Hospital, Guangzhou, China, patients were recruited by face-to-face contact. All eligible patients should be at least age 18 years, cognitively and verbally intact, and willing to participate. Patients who were on temporary hemodialysis or had mental disorders or hearing or speaking disabilities were excluded. In addition, we recruited nephrology healthcare providers and general population from the national network by snowball sampling (sending emails and WeChat Moments). The eligibility criteria for participation include nephrology healthcare providers who were: (1) at least 18 years of age; (2) engaged in the field of nephrology; (3) capable of independent thinking, listening, speaking, reading and writing; and (4) willing to participate; and included the general population aged 18 years or older, working in non-medical occupations, and not suffering from any kidney disease. Data collection was conducted from December 2020 to May 2021.

### Ethical approval and consent to participate

This study has been ethically approved by the Institutional Review Board (IRB) of Jinan University (JNUKY-2020-006). All participants were informed about the study’s objective, scope, and research design, and they consented to participate in the study.

### Questionnaire and DCE design

**Figure 1:**
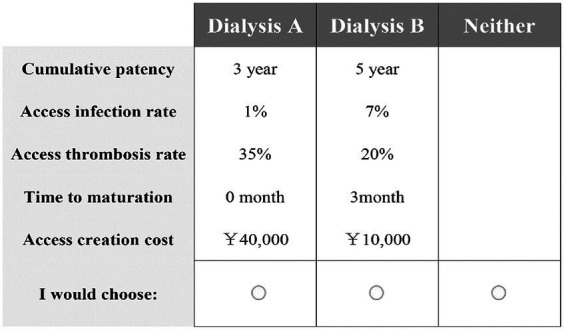
An example discrete choice experiment question.

The questionnaire consists of two sections. The first section is sociodemographic questions on participants, including age, sex, education background, family monthly income, and medical insurance reimbursement rate. The second part is a set of 12 DCE questions designed by Sawtooth Software (version 9.8.1) using an orthogonal experimental design in a partial factorial design, which is an efficient, fast and economical experimental design method. This study used software for orthogonal experimental design in the experimental design session to achieve the three principles of DCE design: Orthogonality, Level balance, Minimal overlap. In a DCE, participants were provided with a dialysis regimen with a combination of different attribute levels in the form of a questionnaire, also known as choice tasks, and each choice task contained three alternatives of hemodialysis treatment: ‘Dialysis A,’ ‘Dialysis B,’ and ‘Neither.’ For each question, participants were asked to choose one of two alternatives that they thought was better by comparing the attributes and levels. Participants were repeatedly asked which alternative they preferred most. Therefore, the DCEs provided information about the relative importance of each attribute and its level. To better control the quality of data, the DCE included a fixed choice question, which is used to ensure that participants understood the questionnaire and made careful choices, controlling the quality of the questionnaire. An example of a DCE question is shown in Figure 1.

Since many patients were over 55 years old with blurred vision, we conducted a discrete choice experiment in the form of face-to-face interviews for patients. Before the survey started, we informed them in detail about the purpose of the study and the meaning of each attribute and level. For healthcare providers with medical backgrounds, we used an online survey to conduct DCE. The purpose of the study and the explanation of each attribute was presented on the first page of the questionnaire.

### Statistical analysis

Participants’ demographic information and socioeconomic status information, and participants’ DCE choices were analyzed three different types of population separately in STATA BE 18 (Stata Corp LLC, TX, United States) using the mixed logit model (MXL). Specifically, the coefficients were calculated to quantify respondents’ relative utility when choosing a specific level within each attribute compared to the reference level. Higher level of the coefficient indicates the specific attribute level was preferred. The core of MXL is the random utility principle (35), and the utility formula that has been applied to estimate our MXL is shown as follows:


$$U_{n}=V_{n}+\varepsilon_{n}=\alpha_{1}+\beta_{1}X_{1n}+\beta_{2}X_{2n}+\ldots +\beta_{m}X_{mni}+\varepsilon_{n}$$

where $U_{n}$ is the total utility of questionnaire respondents, the deterministic component $V_{n}$ is an observable function concerning m levels, the $\beta_{n}$ of $X_{n}$ represents the random utility or the coefficient of every attribute’ levels that bring to the individual relative to the reference level, and $\varepsilon_{n}$ means the fixed utility in our model. The attributes except cost were dummy-coded, and the cost was included as a continuous variable for further analysis. In addition, we calculated the relative importance of attributes by calculating the proportion of one specific attribute to all attributes. We calculated the relative importance using the coefficient difference among a specific attribute, divided by the sum of coefficient differences among each attribute. For instance, in the specific attribute *x*, we use the level with the highest utility ($L_{H}$) to minus the level with the lowest utility ($L_{L}$), and then we use this difference $L_{H}-L_{L}$ to be divided by the sum such differences among all attributes, that is $\sum_{i=1}^{5}{\left(L_{H}-L_{L}\right)}_{i}$.We also calculated the willingness to pay (WTP) of respondents via the multinomial logit model (MNL) by using the continuous variable cost. As an intuitive tool to quantify respondents’ preference for various attribute levels of vascular accesses in the monetary term, WTP is often more intuitive for policymakers and manufacturers.

We have also performed the interaction analysis to investigate whether the demographic information of the participants (especially the general population) interacted with the utility that was brought from the attributes’ levels in the DCE choice tasks. Specifically, we conducted the interaction analysis between age and other attributes, sex and other attributes, education and other attributes, income and other attributes, insurance and other attributes. We also conducted a sub-group analysis based on the interaction terms that were statistically significant in the interaction analysis.

After the interaction analysis and sub-group analysis, we constructed some hypothetical hemodialysis vascular access profiles to investigate participants’ uptake rate when the attributes’ levels were changed compared with the base case profile, which was composed of the reference levels of all attributes.

## Results

### Respondents’ demographic information

After excluding incomplete survey data and data quality controlling by identifying the responses of fixed task choice scenarios, a total of 837 respondents have been included in our study, inclusive of 253 MHD patients, 316 healthcare providers, and the remaining 268 respondents categorized as the general population. Their social-demographic characteristics have been summarized in Table 2. Among those patients, participants had a mean age of 62.5 ± 15.7 years, 110 (44.89%) of them were female, and only 16.40% achieved academic degrees higher than a bachelor’s degree. While among those healthcare providers, 199 (62.97%) of them were female, and the majority (99.05%) have achieved academic degrees higher than bachelor’s degrees. Among the general population, 166 (61.94%) were female, and 240 (89.55%) of them have achieved academic degrees higher than bachelor’s degrees.

**Table 2:** Participants’ demographic information.

Variable	MHD patients (*n* = 253)	Healthcare providers (*n* = 316)	General population (*n* = 268)
Age, mean (*SD*)	62.5 ± 15.7	36.5 ± 8.1	32.2 ± 10.8
Gender, %			
Male	139(54.9%)	117(37.0%)	102(38.1%)
Female	114(45.1%)	199(63.0%)	166(61.9%)
Education level, %			
Less than high school	146(57.7%)	0	8(3.0%)
High school	66(26.1%)	3(1.0%)	20(7.5%)
Any college	41(16.2%)	313(99.0%)	240(89.5%)
Monthly family income, %			
CN¥ <5,000	93(36.7%)	21(6.6%)	61(22.8%)
CN¥ 5,000–10,000	86(34.0%)	133(42.1%)	95(35.4%)
CN¥ 10,000–15,000	46(18.2%)	84(26.6%)	53(19.8%)
CN¥ >15,000	28(11.1%)	78(24.7%)	59(22.0%)
Payment, %			
Fully reimbursed	22(8.7%)	4(1.3%)	9(3.3%)
Urban Employee Medical Insurance	117(46.3%)	296(93.7%)	113(42.2%)
Urban Residents Medical Insurance	79(31.2%)	12(3.8%)	98(36.6%)
Off-site Medical Insurance	29(11.4%)	2(0.6%)	18(6.7%)
Paying completely out of pocket	6(2.4%)	2(0.6%)	30(11.2%)

### Model estimates

**Figure 2:**
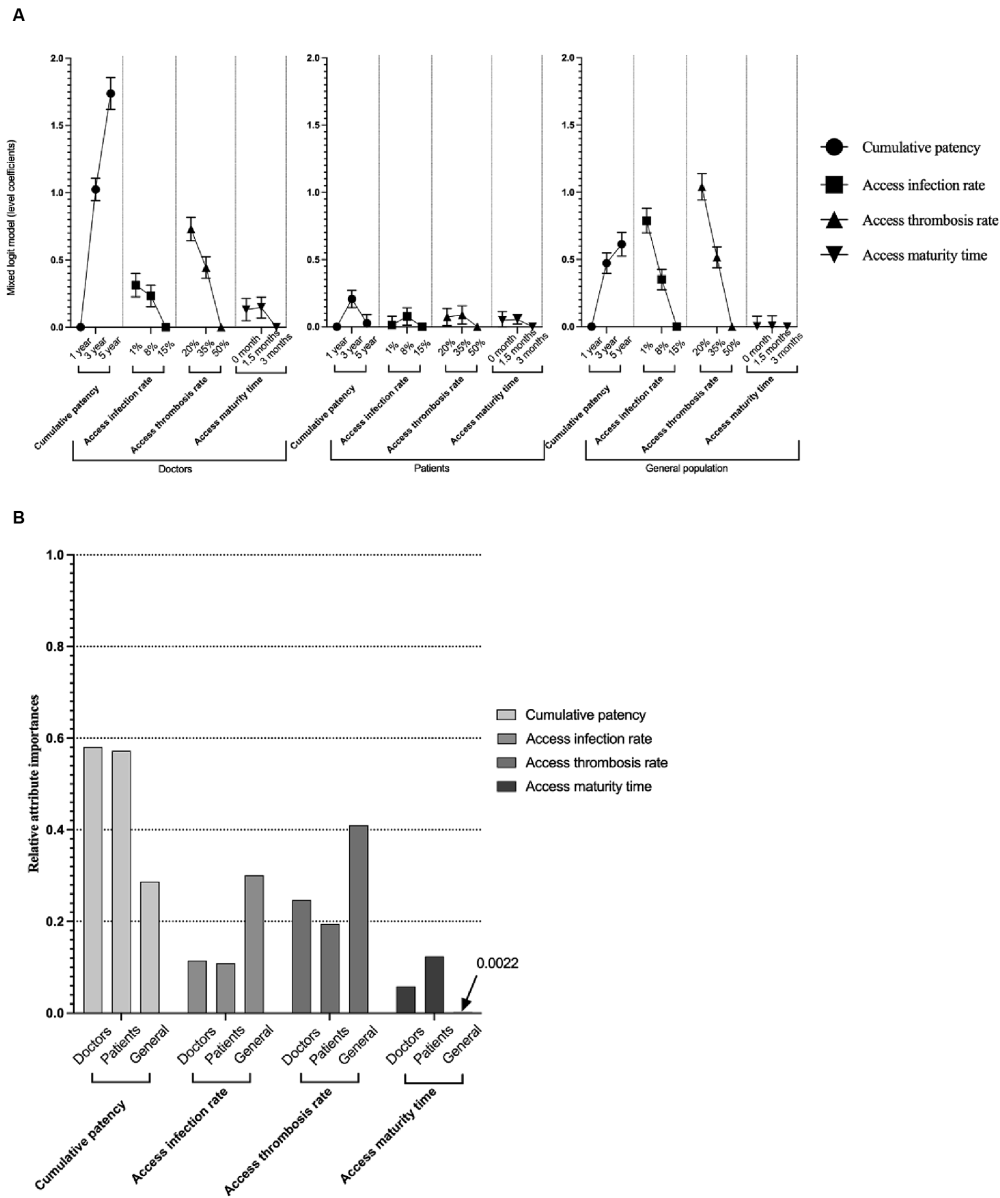
Model estimates of mixed logit model and relative importance of attributes. Panel **(A)** shows the model estimation (coefficient and standard error) of the mixed logit model. All the coefficients in the figure are larger or equal to 0. The order of the Panel **(A)** is the preference of doctors, patients, and the general population. Error bars indicate the standard error of the coefficients, and dots with no error bars mean the reference level of that attribute. Panel **(B)** shows the relative importance of attributes among doctors, patients, and the general population. The maximum value was rescaled to 1.

The model estimates of the MXL model have been shown in Figure 2A. In the healthcare providers and general population groups, respondents always preferred longer cumulative patency, lower access infection rate and lower access thrombosis rate, and respondents’ preference for cumulative patency and access thrombosis rate were sensitive when changing from one level to another level. While in the patients group, 3-year cumulative patency, 8% access infection rate, 35% access thrombosis rate and 1.5 access maturity time were preferred, with only 3-year cumulative patency reaching statistical significance. Respondents in three groups were not sensitive to the attribute access maturity time. Notably, the opt-out option in healthcare providers and the general population group were characterized as a negative sign, indicating negative part-worth utility brought to doctors and the general population (Supplementary Material).

### Relative attribute importance

After rescaling and calculation, in healthcare providers and patients group, cumulative patency was the dominant attribute in respondents’ preference (58.0 and 57.2% respectively), while access thrombosis rate (41.0%) was cared most in general population. Patients care least about access infection rate (10.9%) while access maturity time was cared least in doctors (5.8%) and general population (0.2%). The relative importance of attributes have been shown in Figure 2B.

### Willingness to pay

Patients, healthcare providers and the general population had a strong preference for the cumulative patency within 5 years. Patients’ WTP for cumulative patency was the highest among the three groups of respondents, which was paying RMB¥ 24,720 (US$ 3,708) for each additional year of patency time. Healthcare providers and the general population were willing to pay RMB¥ 15,000 (US$ 2,250) and RMB¥ 10,600 (US$ 1,590) for a 1-year increase in patency time, respectively. Patients and general population had similar WTP for the infection and the thrombosis rates. Specifically, patients were willing to pay RMB¥ 1,200 (US$ 180) to reduce the 1% extra infection rate and up to RMB¥ 2,420 (US$ 363) to reduce 1% of the thrombosis rate. General population were willing to pay RMB¥ 3,730 (US$ 559.5) to reduce the 1% infection rate and up to RMB¥ 2,350 (US$ 352.5) to reduce 1% of the thrombosis rate. Both patients and general population were less sensitive to the wait time for maturation, patients were not willing to pay for a 1-month decrease in time to maturation, and general population were only willing to pay RMB¥ 143 (US$ 21) for a 1-month reduction in time to maturation. However, compared with infection and thrombosis rates, healthcare providers had higher WTP to shorten the maturation time, which was paying RMB¥ 2,900 (US$ 435) for a 1-month decrease in time to maturation. Healthcare providers were less sensitive to the infection and thrombosis rates, and they were willing to pay RMB¥ 1,050 (US$ 157.5) to reduce the infection rate by 1% and RMB¥ 1,150 (US$ 172.5) to reduce the thrombosis rate by 1% (Table 3).

**Table 3:** Willingness to pay of three types of respondents.

Attribute	WTP of patients (*N* = 253)		WTP of Healthcare providers (*N* = 316)		WTP of the general population (*N* = 268)	
	RMB (¥)	USD ($)	RMB (¥)	USD ($)	RMB (¥)	USD ($)
Cumulative patency (per 1-year increase)	24,720	3,708	15,000	2,250	10,600	1,590
Access infection rate (per 1% decrease)	1,200	180	1,050	157.5	3,730	559.5
Access thrombosis rate (per 1% decrease)	2,420	363	1,150	172.5	2,350	352.5
Time to maturation (per 1-month decrease)	−780	−117	2,900	435	140	21
Access creation cost	Ref	Ref	Ref	Ref	Ref	Ref

WTP, willingness to pay. ¥1RMB = $ 0.15USD.

### Interaction analysis and sub-group analysis

In the interaction analysis in the general population, we found that, among the participants’ demographic information, only education level and sex were correlated with our attributes, especially the attribute access thrombosis rate attribute and 20% level (Supplementary Table S1). The negative sign of the interaction term of education with access thrombosis rate of 20% indicated that compared with below bachelor’s degree participants, those with a bachelor’s degree or above have disutility when choosing access with a 20% thrombosis rate.

The sub-group analysis based on the educational level has been presented in Supplementary Table S2. Specifically, we found a statistically significant decrease in utility when choosing access thrombosis rate of 20% for participants with higher educational levels compared with those of lower educational levels. In addition, participants with higher educational levels have similar acceptability for access infection rates of 1 and 8%.

### Uptake rate analysis results

We have presented the uptake rate analysis results in Table 4. In terms of the general population, 79.2% (SE: 0.026) would support the improvement of cumulative patency from 1 year to 3 years, decrease access infection rate from 15 to 8%, decrease time to maturation to 15 months and decrease the access thrombosis rate to 35%. And 92.0% (SE: 0.014) will prefer the improvement of cumulative patency to 5 years, decreasing access infection rate from 15 to 1%. decrease time to maturation to 0 months and decrease the access thrombosis rate to 20%.

**Table 4:** Estimated uptake rate of hypothetical profiles compared with based case scenario.

General population	Profiles of access	Base profile	Profile 1	Profile 2	Profile 3	Profile 4	Profile 5	Profile 6
	Cumulative patency	1 year	3 years	3 years	3 years	3 years	5 years	5 years
	Access infection rate	15%	8%	1%	8%	8%	8%	1%
	Access thrombosis rate	50%	35%	35%	20%	35%	35%	20%
	Time to maturation	30 months	15 months	15 months	15 months	0 month	15 months	0 month
	Estimated uptake of hypothetical profile, No. (SE)	NA	0.792 (0.026)	0.855 (0.021)	0.866 (0.020)	0.792 (0.026)	0.815 (0.025)	0.920 (0.14)

Doctors	Profiles of access	Base profile	Profile 1	Profile 2	Profile 3	Profile 4	Profile 5	Profile 6
	Cumulative patency	1 year	3 years	3 years	3 years	3 years	5 years	5 years
	Access infection rate	15%	8%	1%	8%	8%	8%	1%
	Access thrombosis rate	50%	35%	35%	20%	35%	35%	20%
	Time to maturation	30 months	15 months	15 months	15 months	0 month	15 months	0 month
	Estimated uptake of hypothetical profile, No. (SE)	NA	0.864 (0.020)	0.872 (0.019)	0.894 (0.017)	0.862 (0.020)	0.928 (0.013)	0.948 (0.010)

Patients	Profiles of access	Base profile	Profile 1	Profile 2	Profile 3	Profile 4	Profile 5	Profile 6
	Cumulative patency	1 year	3 years	3 years	3 years	3 years	5 years	5 years
	Access infection rate	15%	8%	1%	8%	8%	8%	1%
	Access thrombosis rate	50%	35%	35%	20%	35%	35%	20%
	Time to maturation	30 months	15 months	15 months	15 months	0 month	15 months	0 month
	Estimated uptake of hypothetical profile, No. (SE)	NA	0.604 (0.031)	0.590 (0.032)	0.601 (0.031)	0.603 (0.031)	0.619 (0.031)	0.599 (0.031)

In terms of doctors, 92.8% (SE: 0.013) will support the improvement of cumulative patency from 1 year to 5, decrease the access infection rate from 15 to 8%, decrease the access thrombosis rate to 35% and decrease time to maturation to 15 months. Also, 94.8% (SE: 0.010) will support the improvement of cumulative patency from 1 year to 5, decrease the access infection rate from 15 to 1%, decrease the access thrombosis rate to 20% and decrease time to maturation to 0 months.

In terms of patients, 61.9% (SE: 0.031) will support the improvement of cumulative patency from 1 year to 5, decrease the access infection rate from 15 to 8%, decrease the access thrombosis rate to 35% and decrease time to maturation to 15 months.

## Discussion

The National Kidney Foundation Dialysis Outcomes Quality Initiative clinical practice guidelines published in 2019 suggest that patient preference should be one of the main considerations in selecting the type of vascular access for hemodialysis (36). Patient preference for different types of vascular access directly affects patient quality of life and the achievement of life goals. Therefore, it is necessary to consider patient preference for different types of vascular access to optimize decisions on vascular access selection, thereby ultimately improving patient treatment satisfaction and prognosis. Vascular access outcomes are key considerations in selecting the best vascular access for an HD patient, including cumulative access patency, type-specific vascular access outcomes, and associated costs (37). This is the first study assessing the preference of MHD patients and nephrology healthcare providers for various hemodialysis vascular access choices focusing on aspects from various attributes of vascular access outcomes. Our results show that MHD patients and nephrology healthcare providers prefer vascular access with longer cumulative patency, which was a prominent attribute in decision-making. Thrombosis rate and infectious rate are also import factors influencing healthcare providers and general population’s decisions. However, respondents were not sensitive to access maturation time. The preference coincides with the characteristics of autologous arteriovenous fistulas (AVF), which have higher long-term patency rates, lower thrombosis rates, and lower costs compared with AVG and TCC. Consequently, it is implied that AVF would be the preferred type of vascular access for MHD patients and health providers based on revealed preference for vascular access attributes.

Cumulative patency is of primary importance for individual patients and healthcare providers, whereas the general population viewed thrombosis events as the most important attribute. This is understandable because, in the long-term clinical treatment process, patients accumulate a lot of knowledge about vascular access since superior patency of vascular access is the pre-conditions and key to ensuring effective hemodialysis treatment. At the same time, the general population has inadequate knowledge about hemodialysis. However, regarding cumulative patency, the general population seems to show a more similar pattern (of the coefficient and *p*-value) to the healthcare providers (of the coefficient and p-value), with preference increasing as the duration of cumulative patency increases. This may make sense because the general population is more well-educated in our sample (Table 2). Most patients are in the “less than high school” category.

In addition, the thrombosis rates also play a significant role in decision-making. Thrombosis often leads to additional surgical interventions to maintain the patency and use of vascular access. These frequent interventions can lead to reductions in patient quality (e.g., discomfort/pain, inconvenience) and increase health care expenditures and, more seriously, most hemodialysis vascular access dysfunction (in both AVF and AVG) is due to stenosis and thrombosis, secondary to venous neointimal hyperplasia (38, 39). For health providers and the general population, infection rates are less important in decision-making than thrombosis rates, possibly because thrombosis is more likely to develop than infections during long-term dialysis (40). In a study focused on investigating patient-reported viewpoints of access-related problems, 97% of patients did not consider infection a major concern (33). Another study suggests that this is because patients believe that the infection is not life-threatening and that even if the catheter is infected, it can be resolved by replacing it with another catheter (34).

The previous literature has reported that income has a significant impact on the choice of initial vascular access in patients with ESKD, which was consistent with our findings. According to a prospective cohort study, total costs of MHD patients are largely determined by treatment requirements rather than by individual characteristics of patients, such as age, sex, social class, diabetes mellitus, hypertension, or time on dialysis (30). It is interesting to note that in our study, the importance of cost consideration for nephrology healthcare providers is also relative high compared with MHD patients. As we learned from the qualitative interviews with nephrologists, the limitations on the total hospitalization cost of each patient in medical insurance policy have resulted in doctors’ attempts to control the cost of treatment. Therefore, their medical decisions are often influenced by costs. The results of the general population were presented as reference. The significance of including the general population in our study is to compare whether they differ from patients in their selection preferences. Compared with doctors and patients, the rate of thrombosis, the rate of infection are more important than the cumulative patency during the decision-making of the general population. This discrepancy may be due to a lack of real-world experience of dialysis. This means that patients newly starting hemodialysis need to be educated about dialysis to increase patient compliance.

## Strengths and weaknesses

While previous cross-sectional studies investigated vascular access preferences, focusing on attributes of patient characteristics. The factors underlying patient’s decisions varied across the countries and races, but consistently important factors were patients’ previous experience with different vascular access, their health status, their desired quality of life, as well as life goals (37). To date, there has been a lack of research comparing preferences for hemodialysis vascular access. This study is the first to use a discrete choice experiment to analyze the importance of vascular access characteristics, which help to provide information for the decision-making process in clinical pathway selection. Our study also provides new insights into how MHD patients and nephrologists make trade-offs between these attributes when making dialysis choices. The US Renal Physicians Association advocates shared decision-making around the initiation of renal replacement therapy (41), which has been described as a process in which physicians and patients agree on a treatment strategy based on a shared understanding of the treatment goals and the risks as well as benefits of the type of treatment chosen (42).

Our results of HD patients were derived from a single health system and not inclusive of the overall picture of HD patients. The study has a limitation in terms of sample representativeness, which could result in selection bias. In terms of the number of attributes, we only included five attributes to describe the characteristics of vascular access to reduce the complexity of the questionnaire and the burden of a questionnaire for respondents. However, other characteristics that will also affect people’s choice, such as patients’ previous experience with different vascular access, their health status, their desired quality of life, and life goals, were not included. Therefore, the advantage of arteriovenous graft (AVG) over tunneled-cuffed catheter (TCC) is not clear due to the limited attributes included in this study. Moreover, we designed the DCE task choices in this study to be unlabeled DCE, since the study of de Bekker-Grob et al. (43) has discussed that although the label of DCE plays an essential role in an individual’s choice, and gives an more realistic feeling, it reduces the attention respondents give to the attributes. While we want respondents to focus more on attributes instead of appearance, etc. However, this DCE does not factor in the influence of the label; therefore, it may result in omitting such influence. Future research may be required to include such label effect in the consideration.

## Implications

These findings emphasize the need for further systematic and longitudinal studies into evaluating vascular access’s cumulative patency and thromboembolic events. The results also indicate that both physicians and patients are most concerned about cumulative patency time, but physicians are more concerned about infection and thrombosis rates than patients. Physicians should consider the patient’s concerns and take the patient’s opinion into account when making decisions. Future research should already investigate other factors that may influence patient decision making and incorporate theory to explore the reasons for differences in treatment preferences between patients and health providers.

## Conclusion

Our study showed that when treating MHD patients, nephrology healthcare providers had a strong preference for access with superior patency, lower thrombosis rate, and lower cost. At the same time, the general population preferred access with a lower thrombosis rate, lower infection rate, and longer patency. According to these results, most patients prefer AVF as an appropriate choice for vascular access if vascular conditions and cardiac function allow it. With the demographic characteristics of the hemodialysis population changing in recent years, it is necessary to consider patients’ preferences for different vascular access to optimize dialysis decisions to ultimately improve patients’ treatment satisfaction and prognosis.

## Data availability statement

The original contributions presented in the study are included in the article/Supplementary material, further inquiries can be directed to the corresponding authors.

## Ethics statement

The studies involving humans were approved by the Institutional Review Board (IRB) review of Jinan University (JNUKY-2020-006). The studies were conducted in accordance with the local legislation and institutional requirements. The participants provided their written informed consent to participate in this study.

## Author contributions

T-SW, QC, and BH contributed equally to the study design, data collection and analysis, manuscript drafting, and formatting. TaL, JY, YG, and YH contributed to the data analysis and manuscript drafting. TaL, QZ, ZT, TiL, JL, and LY contributed to study design and data collection. CZ, JH, and W-KM contributed to the manuscript reviewing and supervision of the study. All authors contributed to the article and approved the submitted version.
