# Supplementary material for: Patients, healthcare providers, and general population preferences for hemodialysis vascular access: a discrete choice experiment
Source: Front Public Health. 2024 May 9;12:1047769. doi: 10.3389/fpubh.2024.1047769 (PMC11112084; doi:10.3389/fpubh.2024.1047769)
Supplement: Supplementary file 1 [file Table_1.DOCX]

**Table S1. Interaction analysis**

| **Levels** | **Coefficient** | **SE** | ***p*-value** | **95% CI** |
| --- | --- | --- | --- | --- |
| opt out | -0.683 | 0.203 | <0.001 | (-1.082, -0.284) |
| Cumulative patency 3year | 0.380 | 0.070 | <0.001 | (0.243, 0.517) |
| Cumulative patency 5year | 0.503 | 0.079 | <0.001 | (0.348, 0.658) |
| Access infection rate 1% | 0.631 | 0.075 | <0.001 | (0.484, 0.778) |
| Access infection rate 8% | 0.295 | 0.065 | <0.001 | (0.169, 0.422) |
| Access thrombosis rate 20% | 0.904 | 0.094 | <0.001 | (0.729, 1.089) |
| Access thrombosis rate 35% | 0.419 | 0.074 | <0.001 | (0.275, 0.563) |
| Time to maturation 0 month | 0.004 | 0.062 | 0.891 | (-0.117, 0.125) |
| Time to maturation_15 month | 0.010 | 0.062 | 0.936 | (-0.112, 0.133) |
| education_Access thrombosis rate 20% | -0.607 | -0.254 | 0.014 | (-1.105, -0.110) |
| Cost | -0.000012 | 2.38e-06 | <0.001 | (-0.00002, -7.24e-06) |

Note: SE: standard error

**Table S2**. Subgroup analysis based on the education level

| Choice | Below Bachelor's Degree | | | Bachelor's Degree and above | | |
| --- | --- | --- | --- | --- | --- | --- |
|  | Coefficient | SE | 95% CI | Coefficient | SE | 95% CI |
| opt | -0.72* | 0.22 | (-1.151, -0.290) | -0.52 | 0.565 | (-1.623, 0.594) |
| Cumulative patency 3 year | 0.402* | 0.072 | (0.260, 0.543) | 0.187 | 0.255 | (-0.313, 0.688) |
| Cumulative patency 5 year | 0.507* | 0.083 | (0.344, 0.670) | 0.458 | 0.256 | (-0.044, 0.960) |
| Access infection rate 1% | 0.644* | 0.080 | (0.487, 0.801) | 0.551* | 0.209 | (0.140, 0.961) |
| Access infection rate 8% | 0.284* | 0.069 | (0.149, 0.420) | 0.398* | 0.178 | (0.050, 0.746) |
| Access thrombosis rate 20% | 0.907* | 0.095 | (0.721, 1.094) | 0.291 | 0.229 | (-0.157, 0.741) |
| Access thrombosis rate 35% | 0..455* | 0.079 | (0.301, 0.610) | 0.109 | 0.209 | (-0.300, 0.520) |
| Time to maturation 0 month | -0.015 | 0.065 | (-0.144, 0.113) | 0.180 | 0.193 | (-0.198, 0.559) |
| Time to maturation_15 month | -0.016 | 0.065 | (-0.144, 0.112) | 0.205 | 0.208 | (-0.203, 0.613) |
| Cost | -0.000011* | 2.56E-06 | (-0.000016, -0.0000062) | -0.0000183* | 6.80E-06 | (-0.000032, -0.0000049) |

Note: * *p* < 0.05
